# Hernie « guidon »: un type rare de hernie pariétale traumatique

**DOI:** 10.11604/pamj.2016.25.110.10808

**Published:** 2016-10-25

**Authors:** Ousseini Adakal, Harissou Adamou, Ibrahim Amadou Magagi, Moussa Koini, Maazou Halidou, Oumarou Habou

**Affiliations:** 1 Département de Chirurgie et Spécialités Chirurgicales, Centre Hospitalier Régional de Maradi, Niger; 2 Département de Chirurgie et Spécialités Chirurgicales, Hôpital National de Zinder, Niger

**Keywords:** Abdominal traumatic wall hernia, handlebar hernia, omental incarceration, Traumatic parietal hernia, handlebar hernia, omental incarceration

## Abstract

Un patient de 20 ans avec une notion de chute sur le guidon d’une moto remontant à 28 jours, était admis pour douleurs abdominales aux urgences chirurgicales. L’examen clinique retrouvait un point d’impact circulaire au niveau de l’hypochondre gauche avec en regard une tuméfaction douloureuse, irréductible et non impulsive aux efforts de toux. Le diagnostic d’une hernie pariétale traumatique étranglée était posé. L’abord chirurgical par laparotomie médiane mettait en évidence une brèche pariétale avec incarcération d’une partie de l’épiploon qui était nécrosée. L’épiploon nécrosé était reséqué et une raphie de la brèche était réalisée. Les suites post-opératoires étaient simples et le patient sortait à j5.

## Abstract (en)

We report the case of a 20-year old patient with a personal history of fall over his motorcycle handlebar occurring 28 days earlier. He was admitted in emergency surgery with abdominal pain. Clinical examination showed a circular impact area at the level of the left hypochondrium associated with painful swelling, irreducible and with no impulse on coughing The diagnosis of traumatic parietal strangulated hernia was established. The patient underwent surgical treatment using midline laparotomy revealing parietal breach associated with incarceration of a portion of the omentum which was necrotic. The necrotic omentum was resected and the breach was sutured. The postoperative course was simple and the patient was discharged on d5.

## Introduction

La hernie « guidon » ou « *handlbar hernia* » des anglo-saxons est un type rare de hernie traumatique [1–3]. Elle est due à un traumatisme direct d’un guidon ou d’un objet similaire sur la paroi abdominale [2, 3]. De diagnostic facile, elle peut être méconnue et se révéler par des complications [3]. Nous rapportons le cas d’une hernie « guidon » compliquée d’une incarcération omentale chez un patient de 20 ans admis 28 jours après le traumatisme.

## Patient et observation

**Figure 1:**
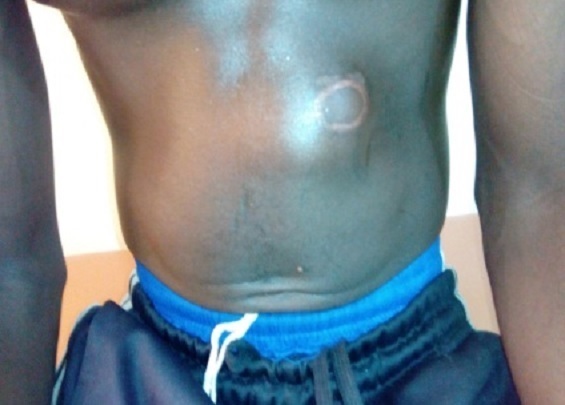
Point d’impact circulaire par le guidon au niveau de l’hypochondre gauche

**Figure 2:**
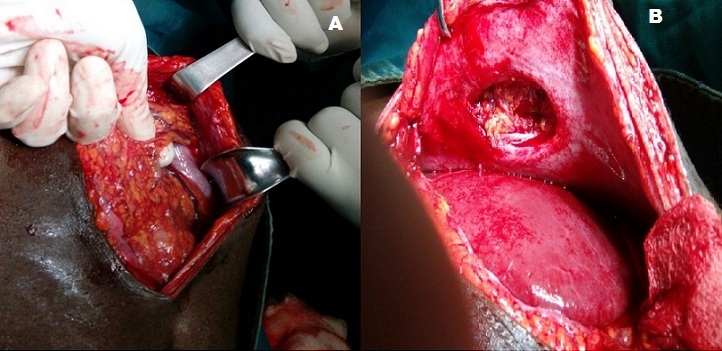
(A) image per-opératoire montrant l’incarcération épiploïque; (B) image per-opératoire de la brèche pariétale

Un patient de 20 ans était admis aux urgences chirurgicales du Centre Hospitalier Régional (CHR) de Maradi pour douleur abdominale aigue. L’anamnèse retrouvait une notion de traumatisme abdominal suite à une chute sur le guidon d’une moto remontant à 28 jours. Le patient avait un bon état général. L’inspection retrouvait un point d’impact circulaire d’environ 4 cm de diamètre au niveau de l’hypochondre gauche, avec des scarifications traditionnelles (Figure 1). La palpation notait une tuméfaction dure, irréductible et douloureuse en regard du point d’impact. Le reste de l’examen clinique était normal. Le diagnostic d’une hernie « guidon » étranglée était posé. Le bilan biologique préopératoire était normal. Une laparotomie médiane avait permis de découvrir une partie de l’épiploon incarcérée et nécrosée dans une brèche pariétale d’environ 3 cm de diamètre (Figure 2). Une résection de l’épiploon nécrosé et une réparation de la brèche pariétale étaient effectuées, par raphie simple plan par plan. Les suites opératoires étaient simples et le patient sortait à J5 postopératoire.

## Discussion

L’incidence des hernies pariétales abdominales traumatiques est faible. Elle est de l’ordre de 1 cas sur 10.000 hernies et de 2 cas sur 3522 accidents [3, 4]. La hernie pariétale traumatique induite par un guidon ou un autre objet similaire a été nommé pour la première fois « handlebar hernia » par Dimayan et al [5] en 1980. C’est une entité rare, qui survient plus fréquemment chez l’enfant et l’adolescent [1, 2, 6–11], rarement chez les personnes âgées [3]. Le mécanisme en cause est un impact focalisé sur la paroi abdominale avec une force suffisante pour entrainer une rupture musculo-aponévrotique, laissant intacte la peau [2, 7, 11]. Les éléments cliniques permettent dans la majorité des cas de poser le diagnostic de hernie « guidon ». Un point d’impact circulaire en regard d’une tuméfaction molle et réductible de la paroi abdominale pose le diagnostic dans les cas simples [2, 10, 11]. Dans certains cas, le patient est admis dans un tableau d’abdomen aigu chirurgical avec un syndrome péritonéal ou occlusif [3, 6, 10, 12]. Toutefois, ces signes cliniques peuvent manquer et la hernie peut passer inaperçue ; dans ce cas, l’imagerie révèle le défect pariétal et d’éventuelles lésions associées [2, 3, 9, 10, 12]. Plusieurs classifications des hernies pariétales traumatiques ont été proposées en fonction de l’objet contondant, de la force du traumatisme, du siège et de la taille du défect [2, 3, 12, 13]. Wood et al [13] rapportait que le traumatisme induit par les guidons et objets assimilés sont plus fréquents et sont la conséquence de forces de faible énergie.

La prise en charge de hernie guidon est chirurgicale. Elle doit se faire dans un bref délai pour éviter l’incarcération ou la strangulation des viscères intraabdominaux [1, 3, 10, 11]. Le geste chirurgical consiste en une laparotomie médiane pour l’exploration du contenu intraabdominal et la herniorraphie ou en un abord électif pour les cas simples [1–3, 10, 11]. Une raphie simple plan par plan est réalisée, cependant dans les larges hernies et/ou chez les personnes âgées, des prothèses peuvent être utilisées [2, 3, 10–12]. La réparation laparoscopique du défect pariétal et/ou des lésions associées a l’avantage d’être moins invasive, sûre et efficace [14]. Devant une instabilité hémodynamique avec un traumatisme sévère, la chirurgie classique est recommandée [2, 14]. L’approche conservatrice peut être proposée en l’absence de lésions intra-abdominales ou d’incarcération de viscères [9, 15]. Le pronostic des hernies « guidon » est en général favorable après un traitement rapide et adéquat [3, 11, 12]. Le retard de consultation, la méconnaissance de cette lésion par les praticiens due à l’absence de symptômes à la phase initiale sont autant de facteurs qui peuvent retarder le diagnostic et grever le pronostic [1–3, 11].

## Conclusion

La hernie guidon est une entité anatomo-clinique rare. Son diagnostic est le plus souvent clinique, mais peut être retardé lorsque la symptomatologie initiale est frustre. Le traitement chirurgical par raphie simple donne de bons résultats.
